# Strategy for Nonenzymatic Harvesting of Cells via Decoupling of Adhesive and Disjoining Domains of Nanostructured Stimulus-Responsive Polymer Films

**DOI:** 10.1021/acsami.3c11296

**Published:** 2023-10-12

**Authors:** Yongwook Kim, Ummay Mowshome Jahan, Alexander Pennef Deltchev, Nickolay Lavrik, Vladimir Reukov, Sergiy Minko

**Affiliations:** † Nanostructured Material Lab, University of Georgia, Athens, Georgia 30602, United States; ‡ Department of Chemistry, University of Georgia, Athens, Georgia 30602, United States; § Department of Textiles, Merchandising, and Interiors, University of Georgia, Athens, Georgia 30602, United States; ∥ Center for Nanophase Materials Sciences, Oak Ridge National Lab, Oak Ridge, Tennessee 37831, United States; ⊥ Lawrence Livermore National Lab, Livermore, California 94500, United States; # Department of Textile Engineering, Chemistry and Science, North Carolina State University, Raleigh, North Carolina 27606, United States

**Keywords:** stimulus-responsive nanostructured coating, polymer brush, cell harvesting, cell adhesion

## Abstract

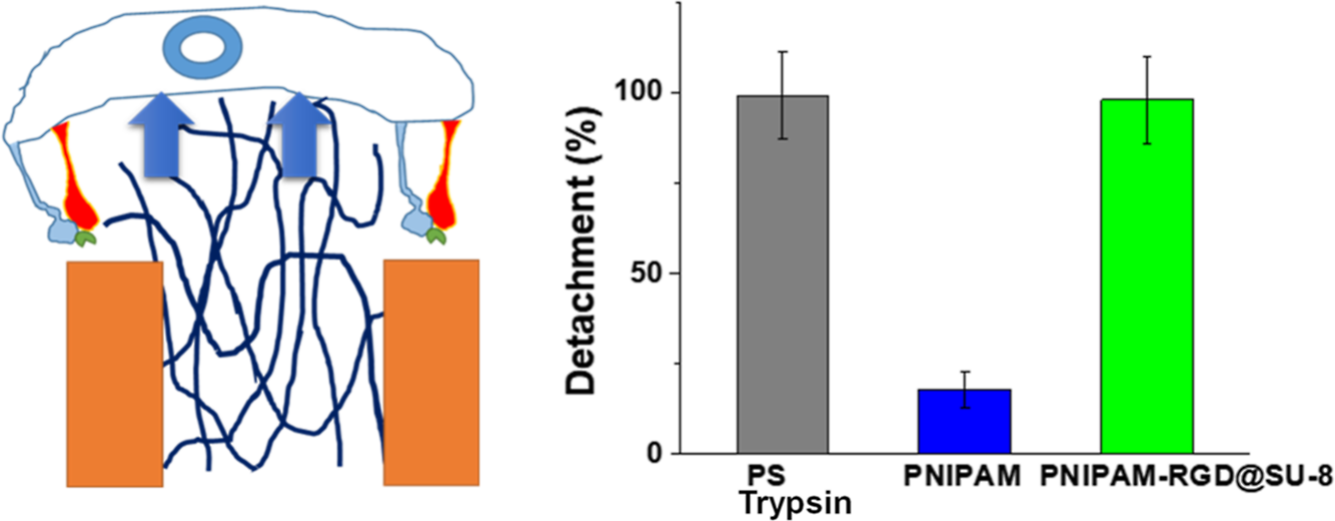

The nanostructured polymer film introduces a novel mechanism of nonenzymatic cell harvesting by decoupling solid cell-adhesive and soft stimulus-responsive cell-disjoining areas on the surface. The key characteristics of this architecture are the decoupling of adhesion from detachment and the impermeability to the integrin protein complex of the adhesive domains. This surface design eliminates inherent limitations of thermoresponsive coatings, namely, the necessity for the precise thickness of the coating, grafting or cross-linking density, and material of the basal substrate. The concept is demonstrated with nanostructured thermoresponsive films made of cell-adhesive epoxy photoresist domains and cell-disjoining poly(*N*-isopropylacrylamide) brush domains.

## Introduction

The nonenzymatic harvesting of live cells became an essential task for various applications in biotechnology of biopharmaceuticals^1−4^ and medicine^5^ amid the rapid growth of the cell manufacturing industry. The major arguments for using nonenzymatic methods refer to the damage done to the membrane and extracellular matrix (ECM) proteins due to the application of proteases for cell detachment and contaminations of the harvested cell culture with the proteases and products of degradation, which are directly related to cell activity potential and functionality.^6−9^ Both these factors impact the applications of the harvested cells.^10^ The arguments against this appertain to different and subtle mechanisms of cell vitiation and shortcomings in harvesting dispersions of cells unbound from ECM materials. The latter statement alludes to the collection and separation of adherent cells grown into cell sheets so that dispersions of cells cannot be obtained without enzymatic cleavage of the ECM, which connects all cells in the sheet. This problem is minimized by the appropriate timing selection for cell harvesting when the confluence does not exceed 80–85%. Possible cell impairments using nonenzymatic methods depend significantly on cell attachment and detachment mechanisms.

A list of alternative cell culture contamination-less methods begins with scraping and includes other mechanical methods that disrupt cell–substrate binding, such as ultrasound,^11−13^ and shear flow.^14^ These methods can be gentle enough to minimize cell damage for many types of cells. However, it is arduous to realize a uniformity of the mechanical force acting to detach the cells over the entire cell growth area. This is due to the dependence of the disjoining force on the location and geometry of the cell culture substrate and the container and the rapid attenuation of the mechanical waves in the medium. These mechanical problems restrain the scalability of the mechanical methods. Chemical and biochemical methods of cell harvesting based on changes in pH of the medium,^15,16^ ion concentration,^17^ electrochemical processes,^18−20^ reversible self-assembled monolayers,^21^ reversible or competitive molecular recognition,^22−24^ and light-induced photochemical processes^25,26^ are limited due to the high sensitivity of cells to the composition of the medium or high costs of the biomaterials. Even minor changes in the medium’s chemical composition, concentrations, and pH can trigger cell apoptosis or mutations.

The most appealing nonenzymatic cell harvesting methods manipulate the temperature-sensitive balance of intermolecular forces to generate disjoining pressure that is applied to the adhered cells.^27−30^ The most used and commercialized (Nunc Dishes with UpCell Surface) example introduced by Okano et al.^31^ is a poly(*N*-isopropylacrylamide) (PNIPAM) coating on glass or plastic plates. This coating undergoes swelling–shrinking transitions when the temperature rises above the lower critical solution temperature (LCST) of 32 °C. Coating-bound and incubated at 37 °C, the cells can be detached from the coating at room temperature. For the best coatings, it takes 5–10 min to detach single cells after lowering the temperature. Since the introduction of PNIPAM coatings for cell harvesting, many different materials with a similar mechanism of temperature response have been developed, including polymer brush architecture, hydrogel coatings, random, block copolymers, or mixtures of PNIPAM with functional monomers or polymers that carry adhesive motifs.^10,27,30−32^ All the proposed architectures have a very similar problem, namely, the necessity of adjusting the characteristic dimension of about 25 nm brought about by the size of the cell binding integrin-adhesive motif complex.

This work proposes a novel concept for efficient nonenzymatic cell harvesting from the supporting surfaces for cell culture. The novelty of the developed method is in the decoupling and separation of disjoining and impermeable adhesive domains on the surface. Such separation of adhesive and disjoining functions is beneficial to adjust cell adhesion to any desired level and, at the same time, control the disjoining forces at any desired grade from a slow, delicate process to a rapid disjoining process. This concept is demonstrated for thermoresponsive nanostructured surfaces that combine impermeable cell-adhesive domains and soft thermoresponsive polymer brush domains that generate disjoining pressure to detach the cells at a temperature below the LCST. Decoupling these two functions—adhesion and disjoining—allows for avoiding multiple problems of thermoresponsive coatings used for cell harvesting applications.

## Experimental Section

### Materials and Chemicals

Si-wafers were purchased from University Wafer, Boston, MA, USA. (3-aminopropyl) triethoxysilane (APTES), ascorbic acid (ASCO), α-bromoisobutyryl bromide (BIBB), triethylamine (TEA), copper(II) bromide (CuBr_2_), *N*,*N*,*N*′,*N*″,*N*″-pentamethyldiethylenetriamine (PMDTA), Arg-Gly-Asp (RGD) peptide, and cyclopentanone were purchased from Millipore Sigma and used as received. Sulfuric acid, dichloromethane, and 30% hydrogen peroxide (H_2_O_2_) were purchased from Fisher Scientific and used as received. 97% PNIPAM was purchased from Millipore Sigma and recrystallized in hexane to remove inhibitors prior to the polymerization. Epoxy-based photoresist SU-8 2002 and SU-8 developer were purchased from Kayaku Advanced Materials, Inc. (USA).

RAW 264.7 cell line, resazurin sodium salt (AlamarBlue), Hanks’ Balanced Salt solution (HBSS), phosphate-buffered saline (PBS), lipopolysaccharide (LPS from *Escherichia coli* O8:K27), rhodamine phalloidin, and Hoechst 33342 were purchased from Sigma-Aldrich (USA). MTT (3-(4,5-dimethylthiazol-2-yl)-2 and dimethyl sulfoxide (DMSO) were purchased from Thermo Fisher Scientific. Dulbecco’s modified eagle’s medium high glucose (DMEM), antibiotics and antimycotics, fetal bovine serum (FBS), 0.25% trypsin–EDTA, glutaraldehyde, and Triton X-100 were obtained from VWR International LLC (USA). For cell culture and cell assay, standard T-75 treated flasks, cell culture treated 24-well plates, and flat-bottom cell culture treated 96-well plates were purchased from VWR International LLC (USA). Wheat germ agglutinin and Vybrant DiD were received from Thermo Fisher Scientific.

### Fabrication of the Photomask

GDSII files of the patterns were compiled using the LayoutEditor software (https://layouteditor.com/). The compiled GDSII files were used to write patterns on 5 in. glass photomasks with Cr metalization on the Heidelberg DWL66 tool (Heidelberg-Instruments, GmbH). Masks with exposed photoresist were developed in a CD-26 developer, rinsed in deionized water, dried, and subsequently etched in acid-based Cr-14 chromium etchant for 2 min. After that, the remaining photoresist was removed in hot *N*-methyl-2-pyrrolidone, rinsed with deionized water, and dried.

### Functionalization of the Surface of Si-Wafers with the ATRP Initiator

The Si-wafer was cut into 0.9 × 0.9 cm^2^ square samples and cleaned in piranha solution (3:1 ratio of sulfuric acid and H_2_O_2_) for 30 min (Warning: piranha solutions react violently with organic materials and can cause an explosion; it cannot be stored in a closed container). The cleaned samples were rinsed with deionized (DI) water and ethanol, followed by drying under an argon flux. Then, the samples were immersed in a 2% APTES solution in ethanol for 24 h to functionalize the surface with amino groups. The surface-bound initiator for the PNIPAM polymerization was immobilized by the immersion of the amino-functionalized samples in 2% TEA and 1% BIBB in anhydrous dichloromethane for 3 h at room temperature. The latter step was followed by rinsing with ethanol and drying under argon.

### Fabrication of SU-8 Structures Using Lithography

Prior to the grafting of the PNIPAM brush, the adhesive domains of the cross-linked SU-8 photoresist were fabricated using photolithography. An 11% SU-8 2002 in cyclopentanone was spin-coated on the initiator functionalized Si-wafer samples at 4000 rpm with an acceleration of 500 rpm/s for 40 s. The formed SU-8 film was baked at 65 °C for 2 min and then exposed to UV light (365 nm, 7 mW/cm^2^) for 15 s through the photomask. A postexposure bake of the sample was carried out on a hot plate at 65 °C for 2 min, followed by the development of the sample in the SU-8 developer for 30 s. Finally, the sample was rinsed with 2-propanol and dried under argon. The thickness of the pattern was measured after a hard bake at 125 °C for 30 min.

### Characterization of the Coatings

Nanostructured polymer films were characterized using Multimode Nanoscope MM8 atomic force microscopy (Bruker Co.) to measure the thickness changes in dry and aqueous states. Samples (0.9 × 0.9 cm^2^) were fixed onto the atomic force microscopy (AFM) stainless disk. The tapping mode with the DPN-S probe (Bruker Co, a silicon nitride top with a diameter of 20 nm and a spring constant of 0.35 N/m) was used to scan the surface of the film. For the aqueous state measurement, samples were placed in a liquid cell filled with a PBS buffer. The temperature of the PBS was controlled by placing an MM8 Heater/Cooler (Bruker Co.) underneath the samples. The baseline for the measurements of the film thickness was approached by scratching the polymer film with a needle down to the surface of the Si-wafer.

### Grafting of the PNIPAM Brush

Polymerization of PNIPAM on the samples with the developed SU-8 structures was carried out via the Activator ReGenerated by Electron Transfer Atom Transfer Radical Polymerization (ARGET-ATRP) mechanism, as reported elsewhere.^44^ PNIPAM was grafted only from the area between SU-8 domains by using the surface-bound initiator unmasked after the development of the photoresist. The sample was immersed in a solution of 40 wt % of recrystallized PNIPAM in 300 μL of DI water and 700 μL of methanol; 9 μL of 0.22 M CuBr_2_ and 9 μL of 0.48 M PMDTA were added to the solution. Oxygen was removed by purging the solution with argon. Then, 50 μL of ASCO (0.04 g/mL) was added to the polymerization vial; it was sealed afterward. The polymerization was conducted in a 35 °C water bath for 30 min. The polymerization was stopped by opening the cap, and the sample was rinsed with ethanol to remove the residual monomers and other chemicals. The samples with uniform PNIPAM coatings (no photoresist structures) were prepared by grafting from the ATRP initiator using the same procedures for the initiator immobilization and grafting of PNIPAM as described previously.

### Functionalization with RGD

RGD was conjugated to the SU-8 adhesive domain through the epoxy–amine reaction of the residual epoxy groups on the surface of SU-8. The samples were immersed in an RGD solution (0.125 mg of RGD in 1 mL of PBS buffer, pH 7.4) for 6 h. Finally, the sample was rinsed with PBS at pH 7.4 and sterilized prior to use for experiments with cells.

### Cell Culture Preparation

RAW 264.7 macrophages were cultured in DMEM, supplemented with 200 U/mL penicillin, 200 mg/mL streptomycin, and 10% FBS. The cells were incubated at 37 °C in 5% CO_2_ and subcultured at about 80% confluency.

### Cell Proliferation on the Nanostructured Thermoresponsive Coatings and Controls

RAW 264.7 cells were cultured on the samples of nanostructured thermoresponsive coatings, uniform PNIPAM brush (control), and standard polystyrene substrate (PS) cell culture plates (control). The samples of the culture support materials were placed in a sterile 24-well plate, and then RAW 264.7 were seeded at a density of 2.5 × 10^4^ cells per well with 1 mL of DMEM and 0.1 μg/L LPS. Cells were incubated for 48 h in a 5% CO_2_ humidified atmosphere incubator at 37 °C. Although it was not specially investigated, we did not observe signs of changes in cell phenotype and the effect of the number of passages with a range of 3–7 passages. We did not quantify the proliferation rate but did not observe noticeable changes in the cell proliferation rate on different substrates. The amount of proliferated cells on the PS and nanostructured PNIPAM substrate was comparable to about 25,000 per cm^2^, while on the reference PNIPAM substrate, the cell count was about 10,000 per cm^2^.

### Cell Harvesting and Staining

The samples of the grown cell culture were gently rinsed with warm (37 °C) PBS to remove the nonadherent cells. Then, the samples were stained using Hoechst 33342 nucleic acid stain and incubated for 20 min. Then, images were taken with an IVIS 100 (IVIS Spectrum imaging system), and the number of total cells on the surface was counted. The cells grown on the PS samples were dissociated using prewarmed 0.25% trypsin–EDTA for 5 min. After that, fresh media were added to neutralize the trypsin, and the cells were collected after centrifuging the cell suspension for 5 min at 300 g. The cells grown on the thermoresponsive surfaces were transferred to a sterile 24-well plate containing 1 mL of fresh culture medium, followed by incubation at room temperature for 10 min. The detached cells were collected in 1.5 mL tubes and used for analysis. The detached cells were counted using a Scepter 3.0 hand-held cell counter. Then, the remaining cells on substrates after cell detachment were stained again using Hoechst 33342 nucleic acid stain and incubated for 20 min. After that, images were taken by an IVIS 100, and the number of total remaining cells on the surface of the samples was counted. For each type of material, including controls, the experiments were repeated three times to estimate the statistical error.

### Cell Viability Assays (AlamarBlue and MTT Assays)

The detached cells collected from each sample were treated with AlamarBlue solution (0.15 mg/mL in PBS, pH = 7.4) at 10% volume of cell culture medium and incubated for 3 h at 37 °C. The active ingredient of AlamarBlue is a nonfluorescent compound, resazurin, which is reduced to resorufin, a red and highly fluorescent substance, after entering the living cells. Its concentration is directly proportional to the number of living cells. The fluorescence was measured at an excitation at 540 nm and an emission at 590 nm using a Varioskan LUX Multimode Microplate Reader. For the 3-(4,5-dimethylthiazol-2-yl)-2,5-diphenyltetrazolium bromide (MTT) assay, the collected cells from each sample were treated with MTT solution (5 mg/mL in PBS) and incubated at 37 °C. After 3 h of incubation, the supernatants were aspirated, and insoluble purple-colored MTT product formazan crystals were dissolved in DMSO for 15 min. The amount of produced formazan is directly proportional to the number of metabolically active viable cells. After that, absorbance was measured at a wavelength of 570 nm using a Varioskan LUX Multimode Microplate Reader. For both assays, three repeats were done for all the samples.

### F-Actin Assay

The detached cells collected from each sample were reseeded with a fresh culture medium to a sterile 96-well plate and incubated for 1 h. Then, the cells were fixed with 2.5% glutaraldehyde for 15 min at room temperature. Then, 0.1% Triton X-100 was used to permeabilize the cells, followed by staining with rhodamine-phalloidin in PBS (50 μg/mL) for 30 min in dark conditions to stain F-actin filaments. After washing with PBS, cell nuclei were stained by using Hoechst 33342 (0.1 μg/mL) for 10 min. The fluorescence images were obtained using an IVIS 100 and the area of F-actin was analyzed using ImageJ software.

### Adhesion Test for Harvested Cells

The detached cells were reseeded in a sterile 96-well plate with the culture medium and incubated for 10 min. Then, the medium was replaced with a fresh culture medium, and the adhered cells were counted to evaluate the number of rapidly adhered cells by using an IVIS 100.

## Results and Discussion

### Analysis of Mechanisms of Cell Attachment and Detachment on Stimulus-Responsive Coatings

**Figure 1:**
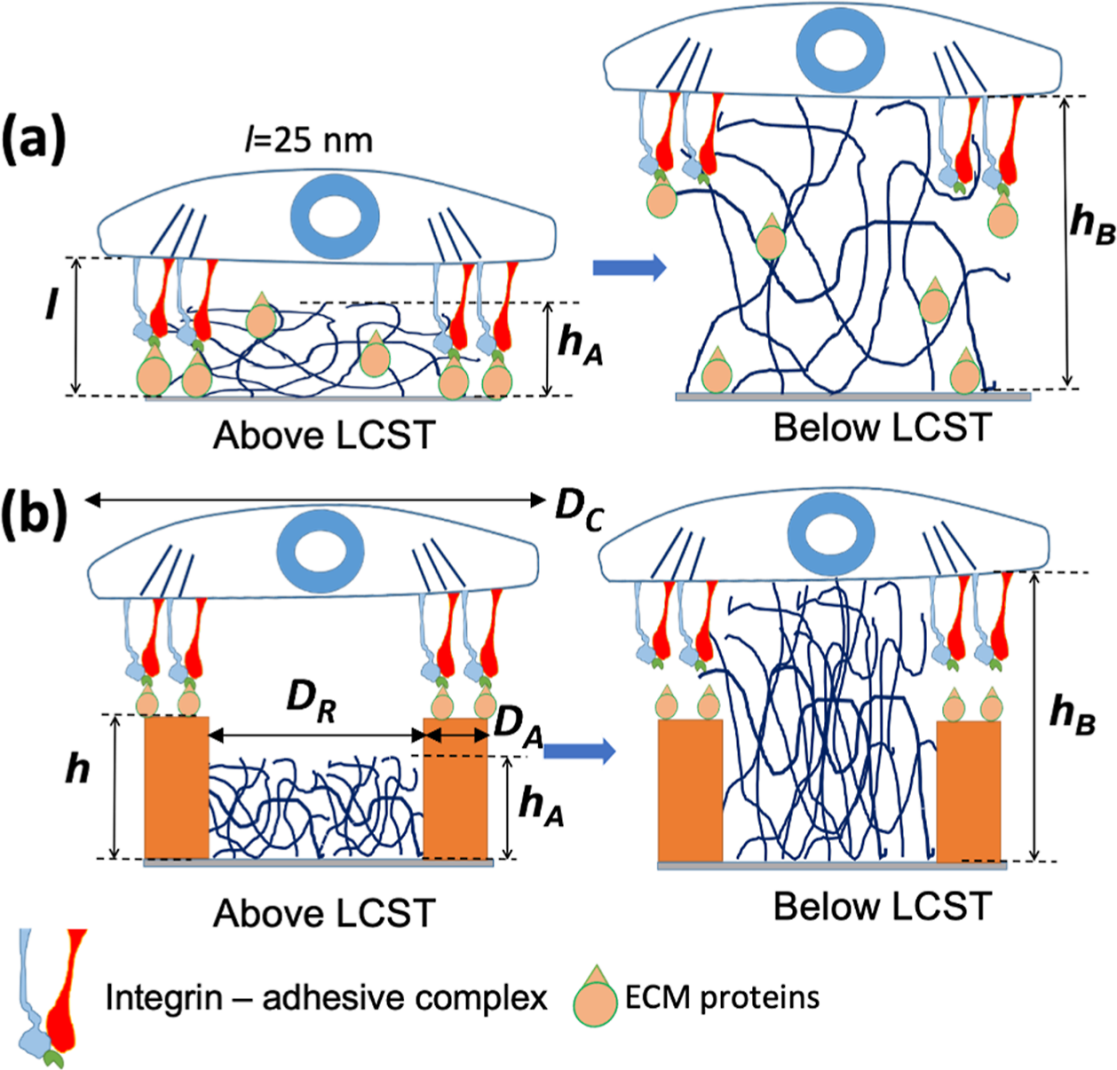
Schematics for enzyme-free cell harvesting mechanism for thermoresponsive cell culture coatings: uniform (a), nanostructured (b), where the critical length scales: *h* is the height of adhesive domains, *h*_A_ and *h*_B_ are the thicknesses of the PNIPAM layer above and below the LCST, respectively; *l* is the length of the integrin protein adhesive complex. *D*_C_ is the diameter of the cell. *D*_R_ and *D*_A_ are the widths of the repulsive and adhesive domains, respectively. *D*_C_ should be bigger than surface features, *D*_R_ and *D*_A_.

Halperin et al. analyzed the mechanism of cell harvesting on thermoresponsive coatings in detail.^33^ Summarized in brief for PNIPAM-based coatings, two different architectures of PNIPAM coatings are used: an end-tethered (grafted) PNIPAM brush^34^ or a cross-liked PNIPAM.^31^ The coating is attached to a glass or a polymeric support in both cases. Cells are seeded on the coating and incubated at 37 °C. First, ECM proteins (e.g., fibronectin) bind to the PNIPAM-coated surface. Alternatively, a NIPAM copolymer with acrylate monomers that carries functional groups (e.g., carboxylic or epoxy) can be further functionalized with a covalently bound polypeptide adhesive motif (typically RGD or ECM proteins).^35^ Then, cells connect to the adsorbed or covalently bound ECM proteins via membrane protein–integrin complexes and form the so-called adhesive focal points. Cells extend and move on the surface owing to the dynamic rearrangement of the cytoskeleton-connected focal points amid the proliferation stage. If the temperature drops below the LCST, the PNIPAM swells, the protein-PNIPAM interactions weaken, and the disjoining pressure applied to the cell membrane builds up (Figure 1a).

One of the three possible mechanisms results in cell detachment: (1) desorption of ECM proteins due to the weakened interactions with PNIPAM, (2) dissociation of adsorbed ECM protein–integrin complexes, or (3) integrin complex push-out of the cell membrane. The first mechanism can be realized if the ECM proteins or polypeptide motifs are not covalently bound to the PNIPAM coating.

The PNIPAM coating structure is critical for cell attachment and detachment mechanisms. ECM proteins adsorb via interactions with the grafting surface (the basal surface of the PNIPAM coating, Supporting Information) or interactions with the PNIPAM chains. Multiple literature reports confirm that the most efficient attachment-detachment of cells was observed for the PNIPAM coating thickness of about *h*_A_ = 20–25 nm at temperatures above the LCST.^36^ Two different mechanisms are discussed to explain this optimal thickness of the PNIPAM coatings. The first hypothesis ponders the major contribution of the grafting surface on ECM protein adsorption.^33^ This fact is explained by the length of the integrin complex of about *l* = 20 nm. Obviously, if *h*_A_ > *l*, then the grafting surface is not accessible for the integrin. The second hypothesis contemplates the vertical gradient of the PNIPAM hydration degree and mobility of the polymer segments.^36^ The hydration degree and segment mobility increase with the distance from the grafting surface. The material of the grafting surface can also affect the degree of hydration via interactions with water and PNIPAM segments. The PNIPAM-water interface has a higher hydration degree as compared to the grafting surface for both the polymer brush and cross-linked coating architectures. A parabolic polymer density profile and stratification of polymer chains of polydisperse PNIPAM explain the higher hydration of the PNIPAM brush–water interface. Cross-link defects and polymer loops and tails explain the increased hydration at the interface of cross-linked PNIPAM coatings. For polymer brushes and cross-linked films, confinement by two interfaces can also contribute to the hydration gradient. The polymer density gradient will affect the dependence of ECM protein adsorption on the PNIPAM coating thickness. Both hypotheses led to similar conclusions about the effect of the grafting surface and the critical role of the PNIPAM layer thickness on ECM protein adsorption.

The cell detachment is related to the ECM adsorption mechanism. Lowering the temperature below the LCST results in the swelling of PNIPAM, which depends on the molecular characteristics of the PNIPAM coating.^37^ It is commonly recognized that the LCST transition is the result of temperature-driven changes in the balance of hydrogen bond energy of the water-swollen PNIPAM. The free energy of equilibrium adsorption of ECM proteins *F*_*pAd*_ is defined by the balance of two terms: the osmotic pressure penalty for the insertion of protein molecules into the PNIPAM layer (*Π_p_*) and the interfacial interaction energy for protein–PNIPAM interaction (*ϒ_p_*):^38^*F*_*pAd*_*(z)* = *Π_p_*(*z*)*V_p_*(*z*) + *ϒ_p_*(*z*)*S_p_*(*z*), where *V_p_* and *S_p_* are the insertion volume and the contact area of the protein molecule fully or partially inserted into PNIPAM, respectively; all are functions of the polymer layer concentration profile (*z*). The osmotic pressure term is lower, while the interfacial energy term is greater for the collapsed PNIPAM at *T* > LCST. Consequently, protein adsorption is promoted at *T* > LCST. The experiments demonstrated much lower adsorption of ECM proteins on the PNIPAM coatings at *T* < LCST. However, once proteins adsorb at *T* > LCST, they do not desorb at *T* < LCST, indicating well-known quasi-irreversible protein adsorption.^39^ ECM protein desorption requires the application of an additional force that originates from the cells bound to the ECM proteins; therefore, this additional force should impact the efficiency of cell detachment.

Two mechanisms are responsible for the force to detach cells. The pull-out force is generated due to the periodic contraction of the cytoskeleton, which is a natural mechanism of cell motility. The second mechanism is the generation of disjoining pressure applied to the cell membrane. The free energy of the cell–PNIPAM interaction can be expressed with a similar equation as for the protein-PNIPAM interaction: *F*_*cAd*_(*z*) = *Π_c_*(*z*)*V_c_*(*z*) + *ϒ_c_*(*z*)*S_c_*(*z*), where the subscript *c* refers to cell. The cell volume is about 10^6^ to 10^9^ greater than the protein volume, while the surface areas differ by a factor of 10^4^ to 10^6^, assuming that the radius of the ECM protein and cell is 9 nm^40^ and 1–10 μm, respectively. Consequently, the disjoining force is substantially greater for cells than for protein molecules. This disjoining push-out force can cause desorption of ECM proteins bound to cell integrin or dissociation of the ECM protein–integrin complex. The dissociation of the protein–integrin complex depends on the direction of the disjoining force vector when faster dissociation rates are observed for the tangential direction of the force.^41^ This mechanism implies restrictions on the PNIPAM coating thickness. If the thickness of the PNIPAM coating at temperatures below the LCST is *h*_B_ < *l*, the swollen PNIPAM will not reach the cell membrane; thus, cell dissociation does not happen.

Importantly, lateral deformation of the polymer brush can affect cell adhesion and detachment and contribute to the effect of optimal brush thickness, as discussed elsewhere.^42^ The consideration of the involved mechanisms and experimental data demonstrates a strong dependence of cell harvesting efficiency of thermoresponsive substrates on the tiny details of the structure of the polymer coatings, first of all, the precise thickness of the coating, grafting or cross-linking density, and material of the basal substrate. Obviously, it also depends on the type of cells because of the different expression levels of the proteins involved in cell adhesion.^43^

The mechanisms discussed previously for cell adhesion and detachment are generally applied to different PNIPAM coatings with a difference in that the adsorption of ECM proteins depends on the grafting density of the polymer brush and on the cross-linked density for the polymer network.

### Concept of Nanostructured Coatings

The concept of the developed stimuli-responsive coating is based on the published results discussed previously that concern the mechanisms of cell adhesion and detachment. The proposed coating here combines two distinct structural domains: impermeable for integrin-cell-adhesive domains and thermoresponsive-cell-disjoining domains (Figure 1b). This design enables the decoupling of the cell adhesion mechanism from the mechanisms generating the disjoining pressure and cell detachment. The surface area of each domain, 4 × 4 μm^2^, is much smaller than the cell projection on the nanostructured coating so that the cell is in contact with several adhesive domains of the height *h*. The adhesive domains are impermeable to integrin. Consequently, there is no effect of the integrin dimension, the adhesive domain thickness, or the concentration profile of the adhesive motifs on cell adhesion. The effect of molecular characteristics of the disjoining brush is also excluded, provided that the brush is thick enough to swell well above the height of the adhesive domains. At the same time, the nanostructured coating mimics the native structured ECM environment.

The lateral dimensions of the adhesive domain in micrometers are sufficient for forming focal points by adherent cells. At *T* > LCST, the PNIPAM domains collapse, and cells are bound to the adhesive domains. At *T* < LCST, the PNIPAM domains swell and develop a push-out force to detach the cells. For this design, only one condition must be met: *h*_A_ ≤ *h* < *h*_B_, where *h* is arbitrarily selected and can have any height above 25 nm to avoid any possible effect on the basal surface. A comparison of the published commonly used architecture of a uniform thermoresponsive coating and the nanostructured coating developed here is illustrated in Figure 1b and in Tables 1 and 2. Importantly, this nanostructured coating concept can be applied to various mechanisms of the response of the domains that develop push-out forces.

**Table 1:** Comparison of Uniform and Nanostructured Stimulus-Responsive Surfaces for Enzyme-Free Cell Harvesting: For Cell Attachment

structural characteristics	uniform coating^33,35,38^	nanostructured coating
grafting density (PNIPAM brush)	low; for adsorption ECM proteins	no limitations^a^
cross-linking density (PNIPAM network)	low; for adsorption ECM proteins	no limitations^a^
thickness of the coating	*h*_A_ < 25 nm	25 nm < *h*_A_ < *h* (*h* is arbitrarily selected)^a^

a Because PNIPAM domains are not in contact with the cell.

**Table 2:** Comparison of Uniform and Nanostructured Stimulus-Responsive Surfaces for Enzyme-Free Cell Harvesting: For Cell Detachment

structural characteristics	uniform coating^33,35^	nanostructured coating
grafting density (PNIPAM brush)	high; to develop disjoining pressure	high; to develop disjoining pressure^a^
cross-linking density (PNIPAM network)	low; to develop disjoining pressure	low; to develop disjoining pressure^a^
thickness of the coating	*h*_B_ > 25 nm	*h*_B_ > *h* (*h* is arbitrarily selected)^44^

a These conditions hold for different designs of push-off mechanisms using polymer brushes and networks.

From the analysis of Tables 1 and 2, we can conclude that in contrast to the nanostructured coating architecture, the precise optimization of the uniform PNIPAM coating’s conflicting characteristics is needed to meet the requirements of cell adhesion and detachment. This precise optimization is difficult to meet at the commercial scales of cell plate fabrication.

We should mention that nanostructured substrates were applied previously for cell cultures to study cell adhesion mechanisms and cell response to the substrate topography as well as for selective cell capture.^45^ For example, nanostructures Si-wafers were reported as substrates to improve the efficiency of cell capture and release for cancer cell assay applications.^46,47^ In those studies, the nanostructured basal surface was uniformly coated by PNIPAM. We recently reported nanostructured surfaces with permeable adhesive and repulsive domains for cell sorting based on their affinity.^44^ However, the design that is based on the decoupling of disjoining and impermeable adhesive domains for cell harvesting is novel.

### Nanostructured Coatings

**Figure 2:**
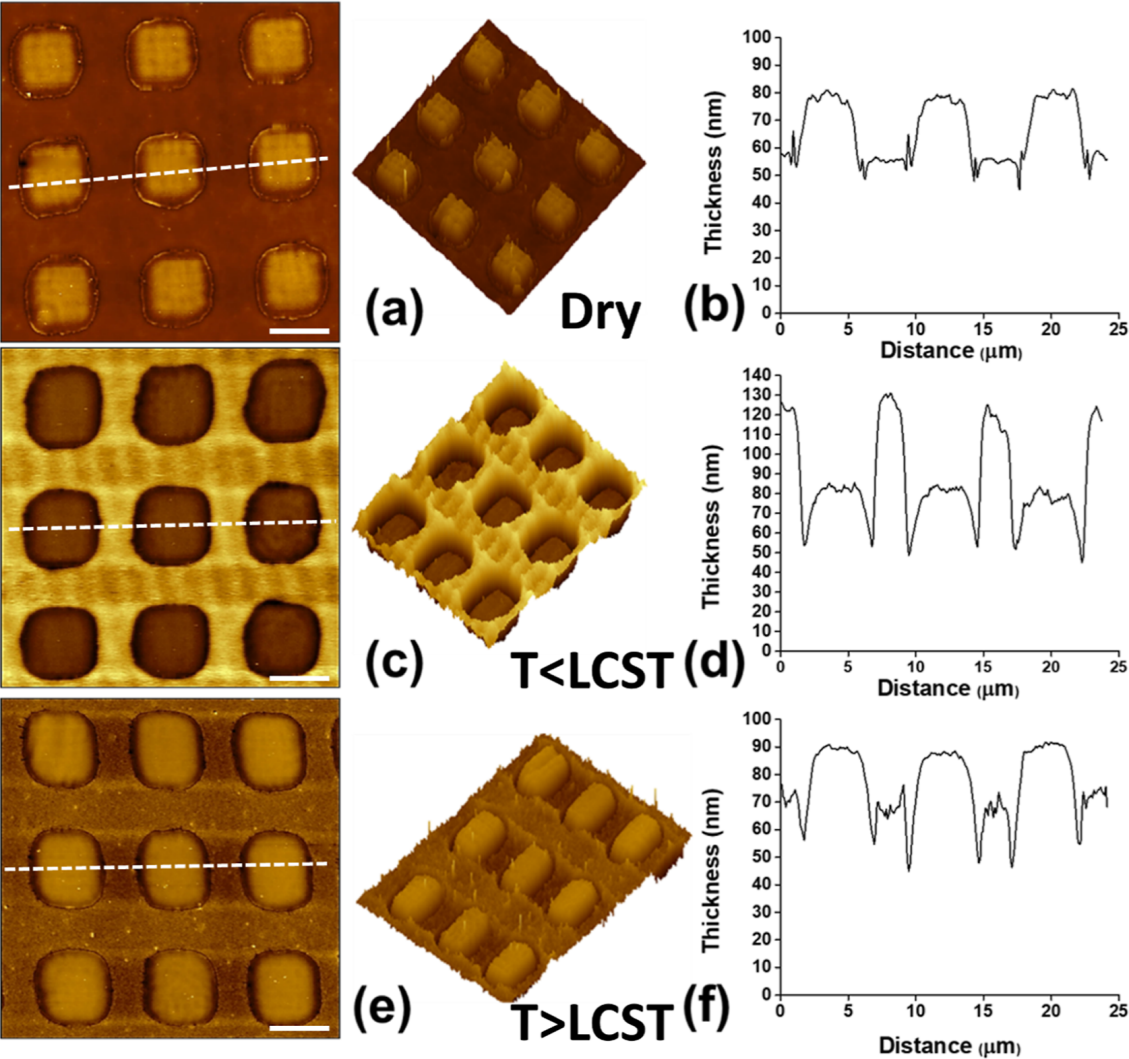
AFM visualization of the nanostructured thermoresponsive coatings with adhesive RGD@SU-8 domains (square “island”) and disjoining PNIPAM brush domains (areas between the islands) is shown, along with corresponding topographical cross sections (b,d,f) in the dry state (a,b). Thickness changes corresponding to the temperature changes of samples in PBS buffer are represented in (c,d) at *T* < LCST and in (e, f) at *T* > LCST. Scale bars 5 μm.

The protocols for the fabrication of the nanostructured PNIPAM coatings, their characterization, and testing in the experiments with cells can be found in the Experimental Section. The nanostructured polymer interface is schematically shown in Figure 1b and Scheme S1a and visualized with AFM (Figure 2) and optical microscopy (Scheme S1b). The coating is composed of two domains representing a 4 μm wide grid pattern geometry. The square shape 4 × 4 μm^2^ and *h* = 80 nm height adhesive domains were fabricated using a SU-8 photoresist and a specially designed photomask (Figure S1). The surface of adhesive domains was conjugated with a cell-adhesive tripeptide, RGD, to make up RGD@SU-8 domains that added 3 nm to the 80 nm thick SU-8 structures. The RGD is a broadly used cell-adhesive peptide sequence that binds to the integrin of the cell membrane.^48−50^

The disjoining domains were made of PNIPAM grafted onto the surface of the Si-wafer substrate. The analysis of the nanostructured films with AFM (Figure 2) performed in several arbitrarily selected areas of the film shows that the SU-8 and PNIPAM brush domains are uniform over the examined surface areas. The accuracy of thickness measurements with AFM is at the subnanometer level. However, the standard error of ±5 nm of the thickness measurements of the domains is the result of the surface roughness of the polymer domains being affected by various film formation conditions. The thickness of the PNIPAM brush in the dry state was 58 (Figure 2a,b), and 68 nm in a PBS buffer at *T* > LCST (Figure 2e,f), while it swelled to a 130 nm thickness at *T* < LCST(Figure 2c,d). Consequently, at *T* > LCST, the RGD@SU-8 domains are 15 nm thicker than the PNIPAM brush, whereas the PNIPAM brush is 47 nm thicker than the RGD@SU-8 domain *T* < LCST. This structure meets the conditions expressed in Tables 1 and 2 when *h*_A_ < *h* < *h*_B_, where *h* = 83 nm, *h*_B_ = 130 nm, and *h*_A_ = 68 nm. For this case, the fraction of the brush that swells greater than *h*, will also swell laterally and cause a tangential force applied to the integrin complex. However, it is difficult to separate the contributions of the tangential and normal forces on cell detachment.

In this work, we did not vary the geometry or dimensions of the domains. These questions should be answered in future studies. For this work, the rectangular shape was selected arbitrarily. The dimensions of the domains, 4 × 4 μm^2^, were selected based on the requirement that the adherent cell (typically 20 μm in diameter before surface binding) should contact both adhesive and disjoining domains for enabling binding and detachment mechanisms. The average size of one focal point is 0.56 ± 0.02 μm^2^, the number of focal points per cell is 65 ± 14, and the total area of the focal points is 36.09 ± 8.45 μm^2^ as published by Horzum et al.^51^

### Efficiency of Cell Harvesting

For the demonstration of the proposed enzyme-free cell-harvesting method, we selected highly adhesive LPS-activated macrophages with enhanced expression of adhesive protein complexes of the cell membrane.^52^

The detachment efficiency of the cell culture from the nanostructured surface was compared with that of the uniform PNIPAM coating and the conventional trypsinization method for the cell culture on the standard PS. A uniform PNIPAM coating was prepared by grafting from the Si-wafer surface using the same protocol for PNIPAM grafting from the nanostructured surfaces. As was discussed above, the optimal PNIPAM thickness should meet the conditions of *h*_A_ < 25 nm and *h*_B_ > 25 nm. For our reference experiment (control), we selected *h* = 83 nm, *h*_B_ = 130 nm, and *h*_A_ = 68 nm, which means *h*_A_ > 25 nm and *h*_B_ > 25 nm. Such a selection was made for two reasons: (i) this PNIPAM coating is of the same thickness as PNIPAM disjoining domains of the nanostructured coatings and hence, we can directly compare the detachment of nanostructured and uniform PNIPAM coatings of the same thickness and corresponding quality of the detached cells; (ii) such a control coating provides a weak cell adhesion because ECM proteins cannot reach the basal surface while the LPS-activated macrophages provide strong binding to the ECM proteins. Therefore, the cell detachment should be realized via ECM protein desorption upon the action of the push-out force, and hence cell damage upon detachment is minimal. In other words, the coating for the reference experiment is beneficial for a delicate cell detachment while out of the optimal characteristics for cell adhesion. To compensate for that, we used cells with enhanced expression of integrin.

**Figure 3:**
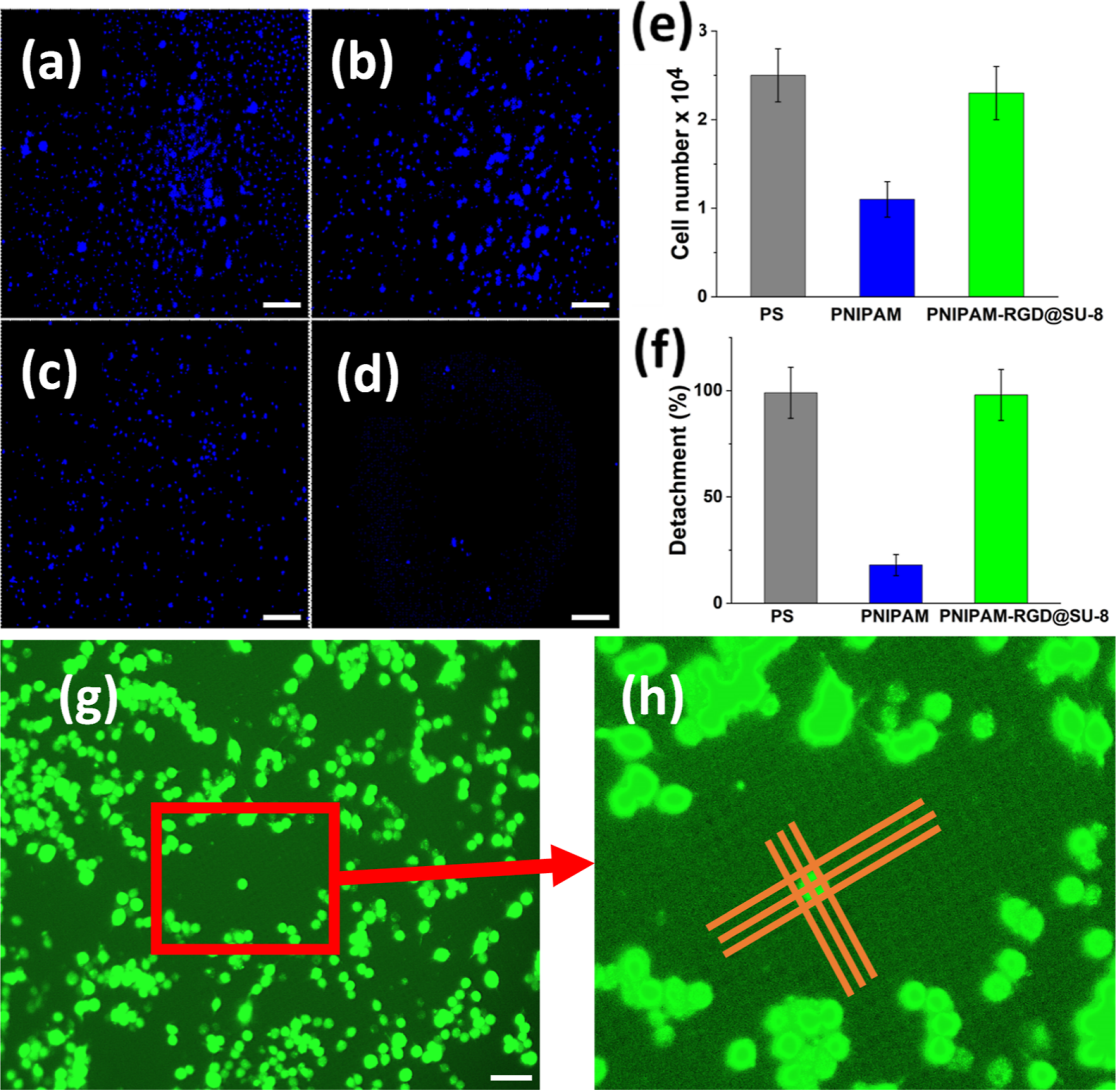
Comparison of RAW264.7 cell culture incubated on the PS control, the uniform PNIPAM brush control, and the nanostructured PNIPAM-RGD@SU-8: fluorescent optical images for the cells on the uniform PNIPAM brush (a, b) and on the PNIPAM-RGD@SU-8 (c,d) at *T* > LCST (a,c) and remaining cells at *T* < LCST (b,d). Counts of the detached cells (e) and fractions of the detached cells (f). Zoomed-up images of the cells residing over several adhesive and disjoining domains (g,h). The orange lines that mark the PNIPAM brush location were drawn for the reader’s convenience. Scale bars are 200 μm.

The LPS-activated RAW 264.7 cells, a monocyte/macrophage cell line, were seeded onto 24-well plates containing the PS substrates (control), the uniform PNIPAM grafted substrate, and the PNIPAM-RGD@SU-8 substrate and cultured in the medium at 37 °C for 48 h. LPS of 0.1 μg/L was added into each well before starting incubation. The attached cells were visualized with an optical microscope (Figure 3). Even the smallest cells are in contact with four adhesive and four disjoining domains of the nanostructured film (Figure 3h). Following the incubation, the cells were collected after lowering the temperature for the PNIPAM and PNIPAM-RGD@SU-8 substrates and by trypsinization for the PS control. For the thermoresponsive surfaces, the cells detached immediately as soon as we approached the temperature below the LCST, typically within 5–10 min. The harvested cells and the cells remaining on the substrate were counted using fluorescent microscopy (Figure 3a–d).

The results of the quantification of the detached cells are shown in Figure 3e,f. It is obvious that the efficiency of cell harvesting (about 98%) by trypsinization and using the thermoresponsive nanostructured film are comparable. The efficiency of the uniform PNIPAM coating (about 17%) is substantially lower despite the fact that the cells were 60% less efficiently bound to the substrate when *h*_A_ > 25 nm. A substantially lower number of cells bound to the uniform PNIPAM coating is evidence of poor cell adhesion. However, even poorly adhered cells were not efficiently detached upon PNIPAM swelling. This result was in good agreement with the recent report by Okano et al. when they did not observe ECM protein desorption from PNIPAM coatings upon cooling below the LCST.^39^ It appears that if cells bind to ECM protein molecules that are adsorbed on the PNIPAM backbone (the secondary adsorption as per classification by Halperin), the detachment condition *h*_B_ > 25 nm is not met. This inefficient detachment is likely due to the translocation of the PNIPAM-connected cells and corresponding swollen PNIPAM segments by the push-out force upward, so the disjoining force is too low to cause cell detachment.

### Viability of Harvested Cells

**Figure 4:**
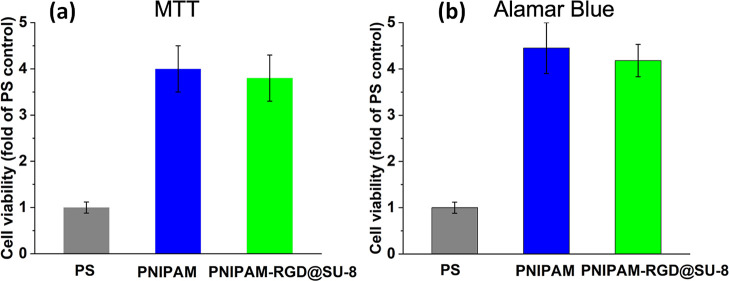
Evaluation of harvested cell viability: MTT assay (a); AlamarBlue assay (b).

The quality of the detached cells (viability) was evaluated by MTT and Alamar Blue assays (Figure 4). These assays are commonly used to assess the metabolic activity of cells. We arbitrarily assigned 100% viability to the control: the cell culture collected from the PS control by trypsinization. The best results were obtained for the uniform PNIPAM brush, and slightly lower viability assay data were obtained for the nanostructured coatings. In both cases, the cell viability characteristics are by a factor of about 4 times better than that of the control. This result is in good agreement with the literature.^31^ Some improvement in cell viability collected from the uniform PNIPAM as compared to that from the nanostructured coating is not fully understood yet. One possible explanation is the cytotoxicity of the metal catalysts used in the SU-8 photoresists. In this work, we did not focus on details related to the cytotoxicity of the materials used for the fabrication of thermoresponsive coatings. The overall concept can accommodate different materials, for example, photo-cross-linkable formulations broadly used for biomedical applications.

**Figure 5:**
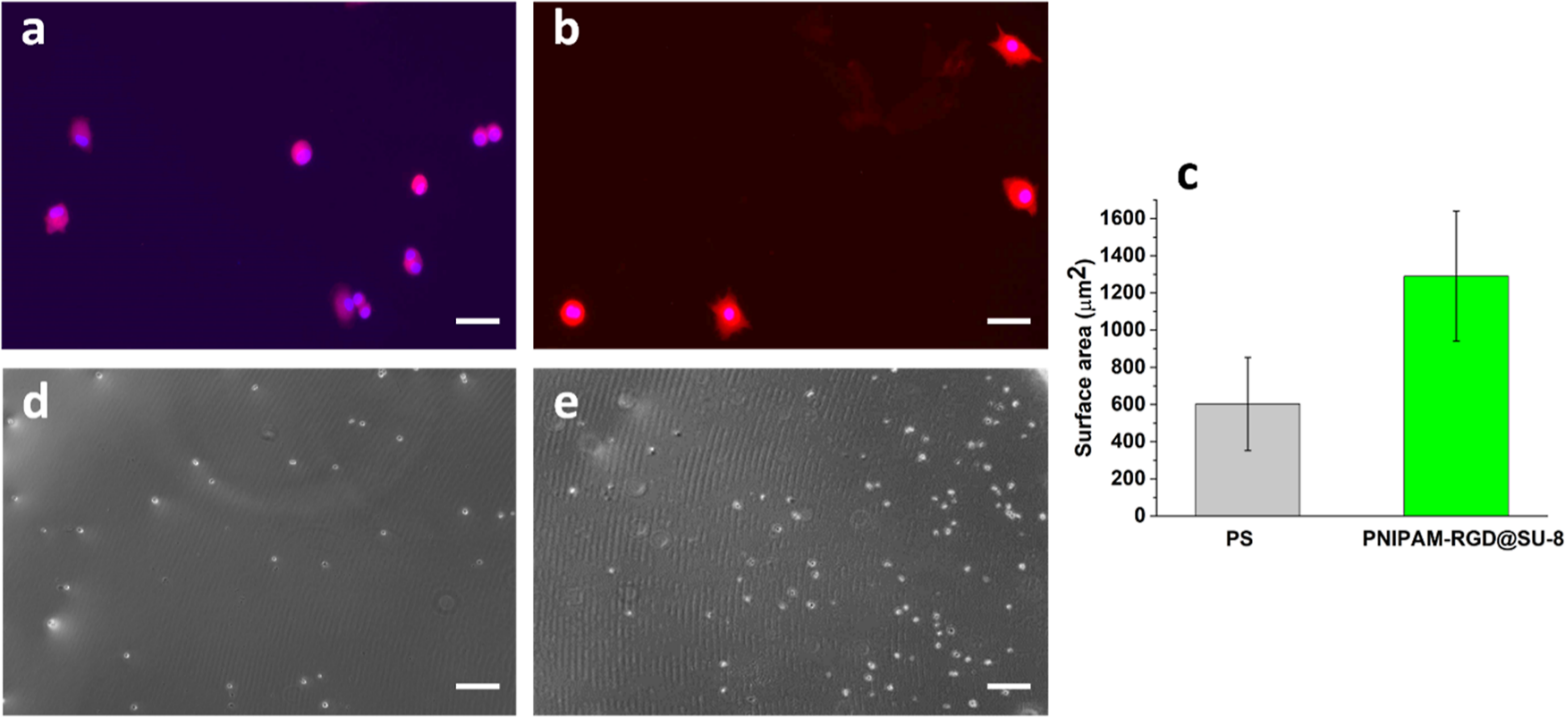
Reseeding of the harvested cells: (a,b) f-actin assay (red fluorescent labels f-actin, blue labels nucleus of the cell), for the cells collected from (a) PS control, (b) PNIPAM-RGD@SU-8; (c) f-actin surface area, (d,e) optical images of the reseeded cells after 10 min incubation time for the cells collected from (d) PS control, (e) PNIPAM-RGD@SU-8. Scale bars (a,b: 25 μm and d,e: 200 μm).

The quality of the harvested cells was evaluated additionally by the ability of reattachment on the surface of the standard PS culture dishes and the comparison of f-actin surface area, where stained with rhodamine-phalloidin in PBS. The same number of cells collected from the nanostructured thermoresponsive coatings and the PS (control) were seeded using the commercial multiwell plates and a fresh culture medium and examined after 1 h incubation time. Unadhered cells were removed by rinsing in PBS buffer. Then, the cells were fixed and stained to label F-actin and the cell nucleus. For the quantitative analysis, we used the fluorescent signal of the stained F-actin to compare the surface areas of the bound cells harvested from the nanostructured thermoresponsive coatings and the control (Figure 5a,b). The surface area of the cells for the first case was twofold greater than that for the control (Figure 5c). For the reattachment experiment, after seeding the same number of cells collected from two methods and waiting for 10 min to let the cells adhere, unattached cells were removed, and fresh PBS buffer was added to count the number of adhered cells. The number of adhered cells that had been collected from the nanostructured thermoresponsive coatings was threefold greater than that for the control (Figure 5d, e). These results are in accord with the viability assays and demonstrate that the membrane proteins are much better preserved by the developed method as compared to the trypsinization method.

## Conclusions

This report shows that the nanostructured thermoresponsive coating is quite an efficient novel approach for nonenzymatic cell harvesting. This method preserves membrane proteins and cell functions similar to other enzyme-free harvesting methods. The advantage of this method is that it is not sensitive to many characteristics that should be precisely optimized in the case of uniform thermoresponsive coatings. This distinction opens a road to much broader applications of such materials in biotechnology. This conclusion is based on the potential scalability of the proposed method by adapting one of the numerous developed approaches to the efficient fabrication of micropatterned polymer coatings. This should be the subject of the following research and developments.

## Supporting Information Available

Optical image of the photomask, schematic of the synthesis of the nanostructured film, and AFM images of the PNIPAM film (PDF)
