# Supplementary material for: Strategy for Nonenzymatic Harvesting of Cells via Decoupling of Adhesive and Disjoining Domains of Nanostructured Stimulus-Responsive Polymer Films
Source: ACS Appl Mater Interfaces. 2023 Oct 12;15(42):49012–21. doi: 10.1021/acsami.3c11296 (PMC10614186; doi:10.1021/acsami.3c11296)
Supplement: Supplementary file 1 — am3c11296_si_001.pdf [file am3c11296_si_001.pdf]

Supporting Information

Strategy to Non-Enzymatic Harvesting of Cells Via Decoupling of Adhesive and Disjoining Domains of Nanostructured Stimuli-Responsive Polymer Film

Yongwook Kim[1,2,5], Ummay Mowshome Jahan[3,6], Alexander Pennef Deltchev[1,2], Nickolay Lavrik[4], Vladimir Reukov[3], Sergiy Minko[1]*

[1]Nanostructured Material Lab, University of Georgia, Athens, GA 30602, USA

[2]Department of Chemistry, University of Georgia, Athens, GA 30602, USA

[3]Department of Textiles, Merchandising, and Interiors, University of Georgia, Athens, GA 30602, USA

[4]Center for Nanophase Materials Sciences, Oak Ridge National Lab, Oak Ridge, TN 37831, USA

[5]Lawrence Livermore National Lab, Livermore, CA 94500, USA

[6]Department of Textile Engineering, Chemistry and Science, North Carolina State University, Raleigh, NC, 27606, USA

*Corresponding author sminko@uga.edu

Table of Contents

Results ………………..……………………………………………………………... 3

References……………………………………………………………………………… 4

RESULTS

The optical images of the fabricated photomask are shown in Figure S1.

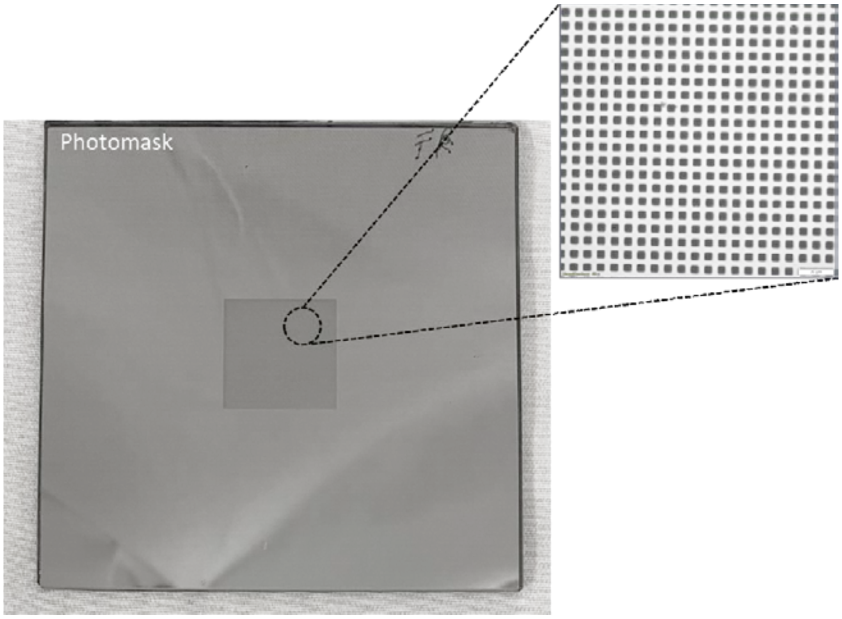

Figure S1. Optical image of the photomask for the fabrication of the nanostructured SU-8 coatings. The scale bar is 20 µm.

The schematic of all steps of the preparation of nanostructured thermoresponsive coatings is shown in Scheme S1.

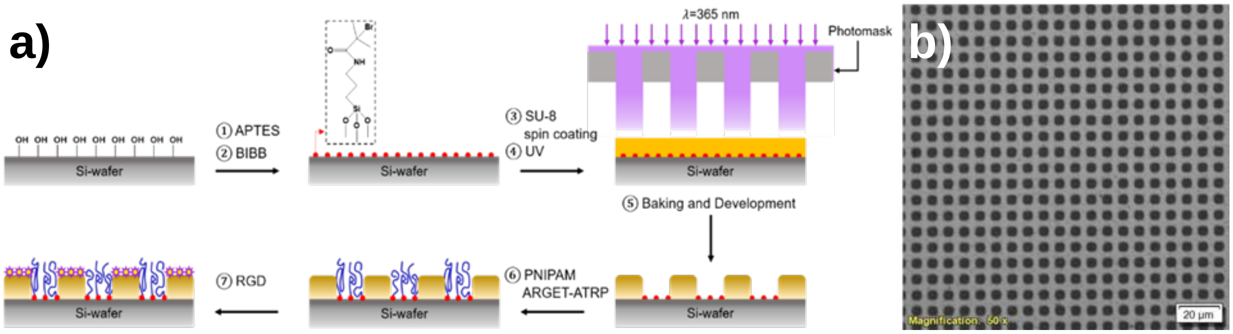

Scheme S1. (a) Schematic of the synthesis of nanostructured thermoresponsive coating: 1,2- Cleaning the Si-wafer in Piranha solution and functionalization with APTES, followed by the immobilization of BIBB - the ATRP initiator; 3,4- fabrication of adhesive domains made of SU-

8 spin-coated photoresist cross-linked under the photomask by a UV light; 5- baking SU-8 film on the hotplate and developing with a SU-8 development solution to produce the patterns; 6 grafting polymerization of NIPAM via ARGET-ATRP mechanism from the surface-bound initiator; 7- conjugation of cell adhesive motifs, RGD to the SU-8 domains.(b) Optical image of the synthesized microstructured coating PNIPAM-RGD@SU8.

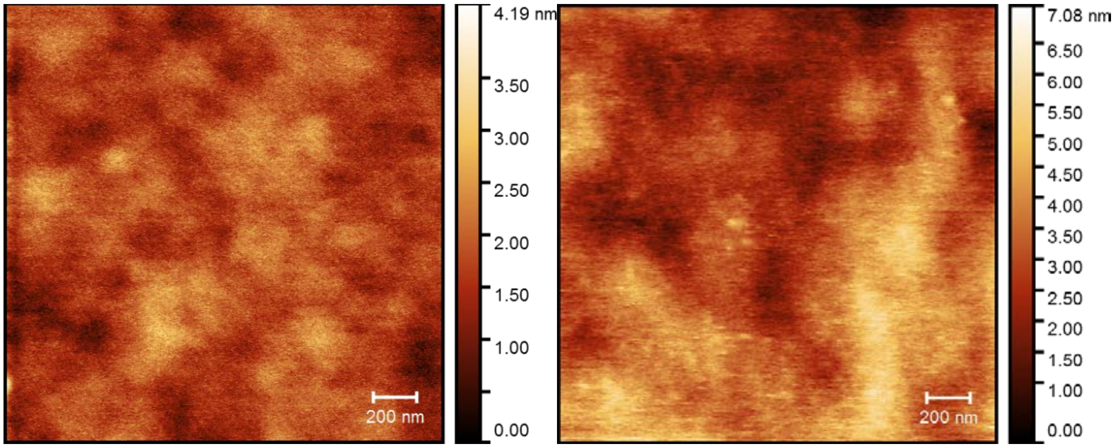

a) **b)**

Figure S2. AFM topographical image of the reference sample of a uniform PNIPAM brush film a) in air with a root mean square roughness of 0.4 nm and b) in water at room temperature with a root mean square roughness of 1 nm. The quantitative analysis of the uniform PNIPAM brush thickness change above and below LCST can be found in our previous publication.[1]

REFERENCES

1. Kim, Y.; Laradji, A. M.; Sharma, S.; Zhang, W.; Yadavalli, N. S.; Xie, J.; Popik, V.; Minko, S. Refining of Particulates at Stimuli-Responsive Interfaces: Label-Free Sorting and Isolation. *Angew. Chem. Int. Ed.* **2022,** *61* (7), e202110990.
